# Acidic pH modulates *Burkholderia cenocepacia* antimicrobial susceptibility in the cystic fibrosis nutritional environment

**DOI:** 10.1128/spectrum.02731-23

**Published:** 2023-11-15

**Authors:** L. Daniela Morales, Yossef Av-Gay, Michael E. P. Murphy

**Affiliations:** 1 Department of Microbiology and Immunology, Life Sciences Institute, The University of British Columbia, Vancouver, British Columbia, Canada; 2 Department of Medicine, Division of Infectious Diseases, Life Sciences Institute, The University of British Columbia, Vancouver, British Columbia, Canada; Georgia Institute of Technology, Atlanta, Georgia, USA

**Keywords:** *Burkholderia cenocepacia*, acidic pH, antimicrobial susceptibility, CF sputum, *in vitro* screening

## Abstract

*Burkholderia cenocepacia* is an opportunistic pathogen correlated with increased disease severity and mortality in cystic fibrosis (CF) patients. One major difficulty when treating these infections in CF is the poor relationship between *in vitro* susceptibility and clinical outcome. Previous analysis of CF sputum samples showed elevated levels of zinc and iron ions and an acidic pH (2.9–6.5) compared to healthy individuals. Additionally, *B. cenocepacia* grows at an acidic pH (~3.5) *in vitro* and persists for 24 h in the intracellular acid compartments of amoebas and macrophages. We seek to understand the impact of acidic pH and increased zinc and iron concentrations on antibiotic susceptibility in the CF nutritional environment. The Synthetic Cystic Fibrosis sputum Media (SCFM) was modified to represent the acidic pH and increased zinc and iron concentrations found in CF sputum (SCFM-FeZn). We found that the nutritional environment modulated *B. cenocepacia* susceptibility to antimicrobials, and more strikingly, acidic pH decreased susceptibility to most antimicrobials used clinically to treat these infections. Finally, we assessed susceptibility against a custom compound library of antimicrobials using SCFM-FeZn agar plates. Out of 591 antibiotics, only 18 were active against *B. cenocepacia* at both neutral and acidic pH. Four of these compounds (novobiocin, coumermycin, mitomycin C, and streptonigrin) were selected for susceptibility assays in liquid culture. Here, we show that acidic pH modulates antimicrobial susceptibility in the CF nutritional environment and that susceptibility testing in media that mimic the host nutritional environment could pave the way to finding new therapies against infections in CF.

**IMPORTANCE:**

*Burkholderia cenocepacia* causes severe infections in cystic fibrosis (CF) patients. CF patients are prone to reoccurring infections due to the accumulation of mucus in their lungs, where bacteria can adhere and grow. Some of the antibiotics that inhibit *B. cenocepacia* in the laboratory are not effective for CF patients. A major contributor to poor clinical outcomes is that antibiotic testing in laboratories occurs under conditions that are different from those of sputum. CF sputum may be acidic and have increased concentrations of iron and zinc. Here, we used a medium that mimics CF sputum and found that acidic pH decreased the activity of many of the antibiotics used against *B. cenocepacia*. In addition, we assessed susceptibility to more than 500 antibiotics and found four active compounds against *B. cenocepacia*. Our findings give a better understanding of the lack of a relationship between susceptibility testing and the clinical outcome when treating *B. cenocepacia* infections.

## INTRODUCTION

*Burkholderia cenocepacia*, an opportunistic pathogen, is highly transmissible and causes severe lung infections associated with increased morbidity and mortality in cystic fibrosis (CF) patients (1, 2). It is intrinsically resistant to antimicrobials and disinfectants, making it hard to treat and eradicate (3, 4). Compared to other *Burkholderia* species that infect CF patients, *B. cenocepacia* is associated with worse outcomes; some patients rapidly develop cepacia syndrome, a necrotizing pneumonia characterized by pulmonary exacerbations, inflammation, and bacteremia, leading to high mortality (5). *B. cenocepacia* survives inside the acid compartments of amoebas, persists in macrophages, and delays phagosomal acidification (6, 7).

Previous analysis of the metal content of CF sputum samples showed elevated levels of iron and zinc compared to healthy individuals (8, 9). Studies measuring the pH of CF airways revealed that the pH is modestly lower in CF than in normal individuals, and more strikingly, direct measurements of sputum samples showed that CF sputum is acidic and can reach pH values of 2.9 (10, 11). Infection and inflammation contribute to sputum acidification (12). During lung infection and inflammation, recruited immune cells can release metabolic acids, and epithelial cells in the airways pump protons and secrete acids that accumulate in the airway surface liquid (13, 14). In addition, bacterial metabolism contributes to infection site acidification. *Pseudomonas aeruginosa* and *Staphylococcus aureus* switch toward anaerobic and fermentative metabolism during infection, releasing byproducts that contribute to reducing the pH of CF sputum and increasing their resistance to antimicrobial therapies (15 – 17).

Environmental pH and metals impact the susceptibility of bacteria to antimicrobials. Acidic pH induces bacterial adaptations associated with antimicrobial resistance and impacts the potency and selectivity of antimicrobial drugs (18 – 20). On the other hand, the presence of metal ions can potentiate the activity of antibiotics and induce the expression of antimicrobial resistance genes (21). To date, there is no standard treatment strategy for *B. cenocepacia* infections in CF patients, and susceptibility testing is not reliable due to the poor relationship between *in vitro* susceptibility to antimicrobials and the clinical outcome (22). We hypothesized that this is because *in vitro* susceptibility assays are often done in rich media, such as Mueller Hinton (MH), which is not representative of the nutritional conditions *B. cenocepacia* is exposed to in the CF lung.

There have been multiple efforts to develop media that better represent the CF nutritional environment. Palmer et al. developed the Synthetic Cystic Fibrosis Sputum Media (SCFM) based on the composition of CF sputum (23). Here, we modified the SCFM to better represent the iron and zinc content and the pH found in CF sputum (SCFM-FeZn). We used the SCFM-FeZn to assess the effect of acidic pH and increased iron and zinc concentrations on *B. cenocepacia* growth and susceptibility to the most commonly used antimicrobials during CF infections. In addition, we assessed susceptibility changes to antibiotics used clinically to treat *B. cenocepacia* infections, and in a set of 591 compounds under more physiologically relevant conditions of pH and iron and zinc concentrations. We found four antibiotics that are active against *B. cenocepacia* at both neutral and acidic pH in the CF nutritional environment.

## MATERIALS AND METHODS

### Bacterial strains and culture conditions

*B. cenocepacia* K56-2 was used in this study. A reporter strain was generated by transforming *B. cenocepacia* with a pBBR1MCS-GFP vector (24, 25). *B. cenocepacia* electrocompetent cells were generated following the protocol previously described in reference (26). For electrotransformation, 100 µL of thawed electrocompetent cells was mixed with 100 ng of vector and incubated for 10 min on ice. The mixture was then pulsed at 2,500 V, and cells were incubated at 37°C for 4 hours in 950 µL of Super Optimal broth with Catabolite repression (SOC) medium. Next, 100 µL of cells was plated on SOC plates supplemented with 50 µg/mL of chloramphenicol (Cm_50_) and incubated at 37°C for 2 days. *B. cenocepacia* cells were freshly transformed prior to each experiment.

For all the growth assays using *B. cenocepacia*, the reporter strain or wild type was grown overnight in MH broth at 37°C. For precultures, overnight cultures were diluted 1:50 into MH and grown at 37°C to an optical density (OD_600_) of 0.4 to 0.6. Cells were spun down and washed in a 0.85% NaCl solution, and the OD_600_ of the cell suspension was normalized before being used to inoculate experimental conditions to an initial OD_600_ of 0.02. *B. cenocepacia* reporter strain precultures and overnight cultures were supplemented with Cm_50_. All OD_600_ and fluorescence measurements were taken using an Infinite 200 PRO Tecan.

### Comparing growth in SCFM, SCFM-FeZn, and MH

SCFM was made as indicated by Palmer et al. (23). SCFM-FeZn was developed by supplementing SCFM based on the average iron and zinc concentrations found in CF sputum described in reference (9, 27). The media were supplemented with iron sulfate and zinc acetate from 10 mM stocks. Iron and zinc stock concentrations were validated using inductively coupled plasma mass spectrometry (ICP-MS). The SCFM buffering capacity was increased by adding 25 mM 3-(N-morpholino)propanesulfonic acid (MOPS) and 25 mM 2-(N-morpholino)ethanesulfonic acid (MES) instead of 10 mM MOPS. The media pH was adjusted using HCl. SCFM, SCFM-FeZn at pH 5.5 and 6.8, and MH were aliquoted into a 96-well plate. The reporter strain was grown as previously described and used to inoculate each media condition. Cells were grown for 32 hours at 37°C. Fluorescence (λ_ex_ = 470 nm, λ_ex_ = 510 nm) and OD_600_ were measured every 2 hours.

### Effect of pH and metals on bacterial growth in the SCFM

SCFM with 25 mM MOPS and 25 mM MES was adjusted to pH 5.5, 5.9, 6.4, and 6.8 using HCl. SCFM at each pH value was supplemented with three levels of iron and zinc, representing the concentrations of sputum from a healthy lung (0 µM Fe, 3 µM Zn), an average CF lung (14 µM Fe, 20 µM Zn), and the highest concentrations found in CF sputum (50 µM Fe, 30 µM Zn). Iron and zinc concentrations were adjusted by adding iron sulfate and zinc acetate together or independently. For the highest concentration of iron found in CF sputum (50 µM), iron was supplemented by adding ferritin from equine spleen (Sigma, F4503-500MG). The iron concentration of the ferritin stock was determined using ICP-MS. One hundred microliters of SCFM supplemented with different levels of iron and zinc and different pH values was aliquoted in a 96-well plate. The reporter strain was grown as previously described and used to inoculate each media condition. Cells were grown for 24 hours at 37°C, and final fluorescence was measured.

### Minimum inhibitory concentration determination

The minimum inhibitory concentration (MIC) was determined in liquid culture using the broth microdilution method in flat-bottom 96-well Corning plates. MICs were determined in liquid MH and SCFM-FeZn at pH 6.8 and 5.5. Briefly, 100 µL of desired media was aliquoted into each well of the 96-well plate using a multichannel pipette. One hundred microliters of antibiotic-supplemented media was then added to the first row. Using a multichannel pipette, antibiotics were serially diluted twofold six to eight times. The medium with no antibiotics was used as a bacterial control.

*B. cenocepacia* K56-2 cells were transformed with the pBBR1MCS-eGFP vector before MIC determination. Precultures were set from overnight cultures, as previously described. Precultures were spun down and resuspended in a 0.85% NaCl solution. This suspension was used to inoculate 96-well plates to an initial OD_600_ of 0.02. Plates were incubated overnight (16 hours) at 37°C. After incubation, fluorescence was measured (λ_ex_ = 470 nm, λ_ex_ = 510 nm).

### Susceptibility assays in SCFM-FeZn plates using the Sweet library

SCFM-FeZn agar plates with pH adjusted to 5.5 and 6.8 were made to assess *B. cenocepacia* K56-2 susceptibility to compounds present in the Sweet library (28). The Sweet library is a 591-compound library that includes most of the commercially available antibiotics. For susceptibility assays, SCFM-FeZn agar plates were made by adding 15 g/L of agar into a media buffer base adjusted to the desired pH and then autoclaved. Sterile amino acids, glucose, lactate, MgCl_2_, CaCl_2_, zinc acetate, and iron sulfate were mixed together and pH adjusted. This solution was added to the autoclaved buffer base and agar solution. After mixing, 45 mL of SCFM-FeZn agar was poured into Nunc OmniTray single-well sterile plates. Plates were left at room temperature overnight.

On the day of the assay, SCFM-FeZn plates were preinoculated with *B. cenocepacia*. SCFM-FeZn buffer base containing 0.7% agar was autoclaved and cooled to 55°C. *B. cenocepacia* precultures were set as previously described, and cells were harvested and washed with a 0.85% NaCl solution. Cell suspension was used to inoculate buffer base + 0.7% agar (initial OD_600_ = 0.05). After mixing, 22 mL of bacterial + 0.7% agar suspension was poured on top of SCFM-FeZn plates. Inoculated plates were left at room temperature for 1 hour until the top layer solidified.

Library compound plates were thawed 1 hour before the assay. Compounds were added to the plates using a BioRobotics BioGrid Robot Microarrayer equipped with a 96-pin tool (0.7 mM diameter pins). After pinning, plates were incubated at 37°C for 24 hours. The inhibition zones of each antibiotic were measured the next morning. Pining was performed in duplicate on two different days (*n* = 4).

### Data analysis

The growth rate was determined from the slope of the growth curve for each condition using the fluorescence data. The maximum growth was defined as the slope during the linear region (*R* ^2^ >0.9) of the exponential phase. For MIC determination, dose-response curves were generated using the Dose-Response Curves (drc) package in R Studio (version 3.0-1) (29). The MIC of each compound was determined using the growth percentage ( growth% ) in the presence of each antibiotic concentration. The percentage of growth was calculated as follows:


$$growth\%=\left(\frac{{FL}_{x}}{{FL}_{0}}\right)x100$$

where *FL_x_* is the fluorescence value at antibiotic concentration *x* and *FL*_0_ is the fluorescence value in media with no antibiotic supplementation.

The packages dplyr and tidyr were used to process data in R Studio, and the ggplot2 package was used to generate boxplots, growth curves, killing curve graphs, and heatmaps.

## RESULTS

### *B. cenocepacia* grows faster and has a higher density in SCFM-FeZn at an acidic pH than in Mueller Hinton and SCFM

Palmer et al. (23) developed the SCFM that mimics the nutritional environment found in CF sputum. However, this medium does not accurately represent the pH or the iron and zinc concentrations found in CF sputum. SCFM has a pH of 6.8, 3.6 µM iron, and no zinc supplementation. The pH of CF sputum samples is measured to be between 2.9 and 6.5, and the pH values at sites of inflammation are as low as 5.5 (10, 30). We modified the SCFM to assess the impact that acidic pH, in combination with iron and zinc, has on *B. cenocepacia* growth. To assess the impact of acidic pH, the buffer base of the SCFM was modified, and the pH was adjusted to 5.5 and 6.8. CF sputum samples have average iron and zinc concentrations of 14.3 and 19.7 µM, respectively (9). To assess the impact of iron and zinc supplementation on *B. cenocepacia* growth, SCFM was supplemented with 14 µM Fe iron and 20 µM Zn zinc. We denominated this medium “SCFM-FeZn.”

**FIG 1:**
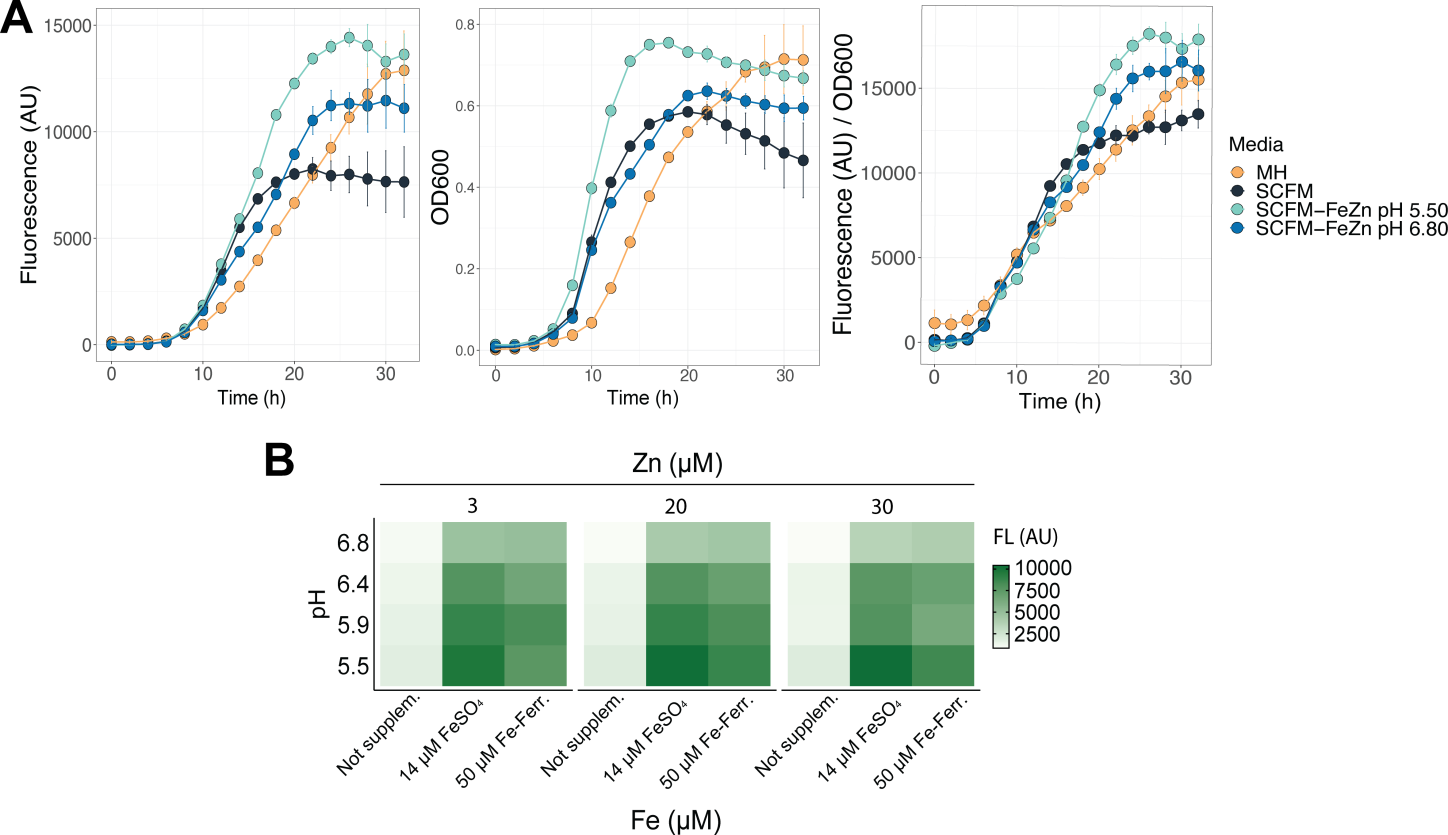
Acidic pH and iron supplementation enhance *B. cenocepacia* K56-2 growth in the CF nutritional environment. *B. cenocepacia* was grown in MH, SCFM, and SCFM-FeZn at pH 5.50 and SCFM-FeZn at pH 6.80 (initial OD_600_ = 0.02). (**A**) Fluorescence and OD_600_ were measured every 2 hours for 32 hours while incubating at 37°C. (**B**) Average fluorescence of the reporter strain after 24 hours of incubation in SCFM at pH 5.5, 5.9, 6.4, and 6.8, supplemented with varying concentrations of iron (not supplemented, 14 and 50 µM) and zinc (3, 20, and 30 µM). Zinc was supplemented with zinc acetate. Iron was supplemented with iron sulfate (FeSO_4_) for 14 µM and with ferritin-bound iron (Fe-Ferr.) for 50 µM. Measurements were taken from three replicates from two independent experiments (*n* = 6).

We generated a reporter strain by transforming *B. cenocepacia* with a plasmid that constitutively expresses eGFP (pBBR1MCS-eGFP). This strain was grown in MH, SCFM, and SCFM-FeZn at acidic and neutral pH. OD_600_ and fluorescence were tracked during 32 hours (Fig. 1A). Optical density and fluorescent measurements were similar during lag and early exponential growth. Interestingly, at higher cell densities, optical density measurements indicated earlier entry into the exponential phase than fluorescence in the SCFM and the SCFM-FeZn. This was likely caused by cell aggregation under these media conditions and suggested that fluorescence was a better indicator of final cell density.

Using the fluorescent measurements, we compared growth by looking at the final cell density and maximum growth rates in the four media conditions. *B. cenocepacia* grew to the highest cell densities in MH and SCFM-FeZn at acidic pH. Cell density increased ~20% and 50% in SCFM-FeZn at acidic pH compared to SCFM-FeZn at neutral pH and SCFM, respectively. We calculated the maximum growth rates on each media condition during exponential growth and found significant differences between all conditions (*PP* < 0.01) (Table S1). The highest growth rate was observed in SCFM-FeZn at an acidic pH and the lowest in MH. In SCFM-FeZn at acidic pH, cells grew ~40% faster than in MH and ~30% faster than in SCFM-FeZn at neutral pH and SCFM. This suggests that acidic pH in combination with iron and zinc supplementation increased growth rate and cell density compared to MH and SCFM.

To further characterize the role of pH, iron, and zinc together and independently on the growth of *B. cenocepacia* in the CF nutritional environment, we supplemented SCFM with iron and zinc levels found in healthy sputum (0 µM Fe, 3 µM Zn), average CF sputum (14 µM Fe, 20 µM Zn), and the highest concentrations in CF sputum (50 µM Fe, 30 µM Zn). Previous research has shown a correlation between increased iron and ferritin concentrations found in CF sputum samples (8). Thus, for the highest iron concentration (50 µM), iron was supplemented by adding equine ferritin. SCFM at pH values ranging from 5.5 to 6.8 was supplemented with the three levels of both metals together or independently (Fig. 1B). After 24 hours of growth, we observed the lowest cell density at the highest pH and no iron supplementation and the highest cell density in media supplemented with 14 µM iron at the lowest pH value. Zinc supplementation did not affect the final cell density. All in all, we found that in the CF nutritional environment, the combination of acidic pH and increased iron enhanced the growth of *B. cenocepacia*.

### Acidic pH modulates *B. cenocepacia* susceptibility to the most commonly used antimicrobials to treat CF lung infections

Environmental pH and the presence of metals can impact the antimicrobial activity of certain compounds. We compared *B. cenocepacia* susceptibility to the most commonly used antimicrobials in MH and SCFM-FeZn at pH 5.5 and 6.8. Since the presence of chloramphenicol used for plasmid selection could impact *B. cenocepacia* susceptibility during the assays, we assessed the fluorescence stability of our reporter strain in the absence of chloramphenicol. There was no significant difference between the fluorescence of the reporter strain grown overnight with and without antibiotic selection (*P* = 0.72) (Fig. S1). Thus, chloramphenicol was not supplemented in the media for susceptibility assays.

**FIG 2:**
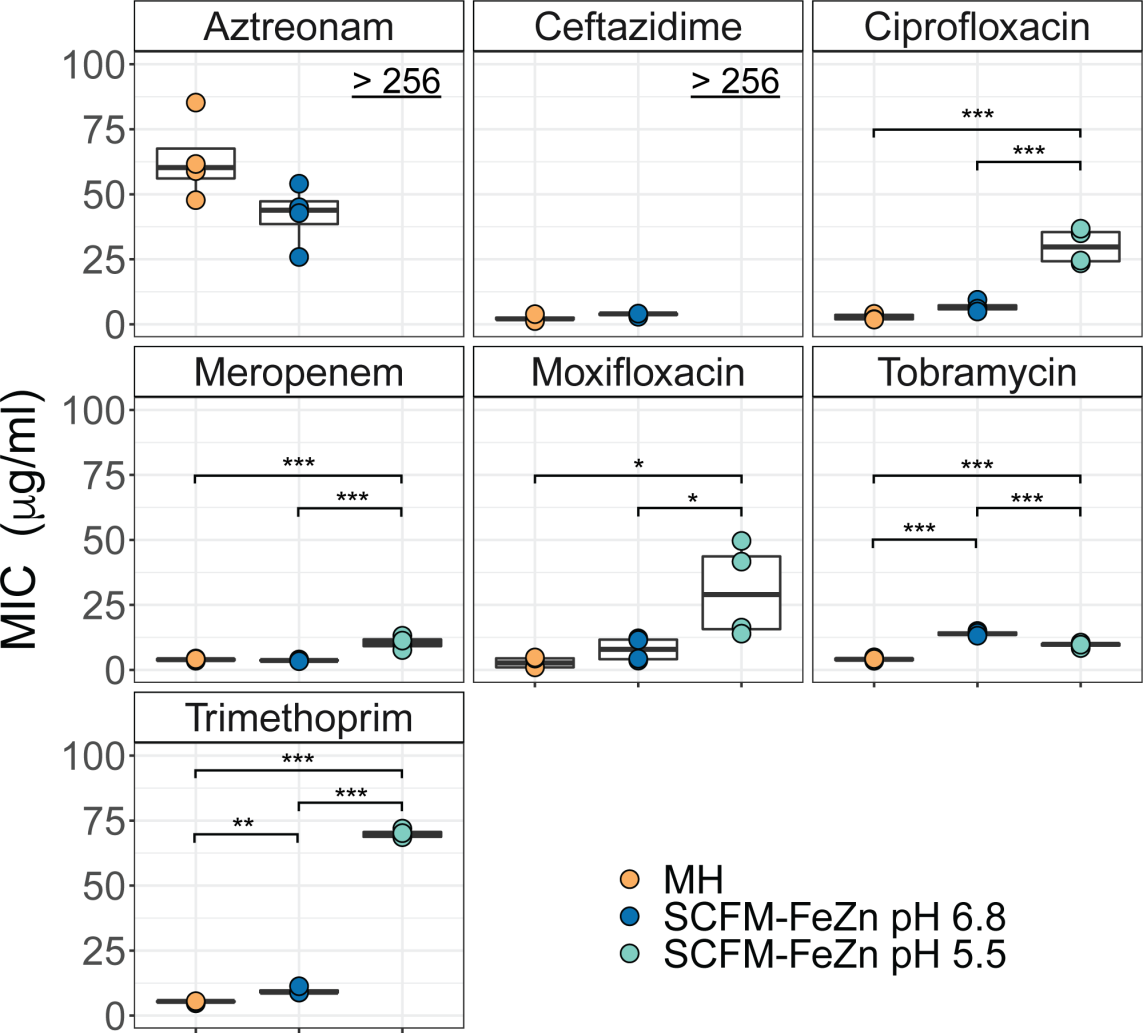
Acidic pH modulates *B. cenocepacia* susceptibility to the most commonly used antimicrobials. The microtiter broth dilution method was used to assess *B. cenocepacia* K56-2 susceptibility to the most commonly used antibiotics to treat CF infections in MH broth and SCFM-FeZn at pH 5.50 and 6.80. The antibiotic concentration that inhibits 90% of growth (MIC) was calculated based on the fluorescence of the reporter strain measured after 16 hours of incubation at 37°C. MIC was calculated independently from data from each of four biological replicates using the drc package in R Studio (*n* = 4). A one-way ANOVA with Tukey honest significant difference in R Studio was used for statistical analysis between MIC values on each medium. Significant differences are indicated as follows: ***P < 0.001, **P < 0.01, and *P < 0.05.

Some of the most commonly used antibiotics to treat *B. cenocepacia* infections in CF patients include aztreonam, ciprofloxacin, meropenem, trimethoprim, moxifloxacin, ceftazidime, and tobramycin. These seven antibiotics target different processes: aztreonam, meropenem, and ceftazidime target cell wall biosynthesis; trimethoprim inhibits folic acid metabolism; moxifloxacin and ciprofloxacin target DNA replication; and tobramycin targets protein synthesis. We measured the fluorescence of the reporter strain incubated with increasing concentrations of these antibiotics and calculated the minimum concentration that would inhibit 90% of growth (MIC) in MH and SCFM-FeZn at pH 5.5 and 6.8 (Fig. 2). We first compared the MIC of the antibiotics in SCFM-FeZn at pH 6.8 and MH. Of the antibiotics tested, a significant decrease in *B. cenocepacia* susceptibility was observed with trimethoprim and tobramycin. The MIC values for trimethoprim and tobramycin were two- and threefold higher in the SCFM-FeZn than in MH, respectively.

We observed more substantial changes to antibiotic susceptibility in SCFM-FeZn at acidic pH than in SCFM-FeZn at neutral pH. Acidic pH significantly decreased *B. cenocepacia* susceptibility to most of the antibiotics tested. Aztreonam and ceftazidime fully failed to inhibit *B. cenocepacia* growth at acidic pH, while the MIC for the majority of antibiotics significantly increased at acidic pH. At acidic pH, the MIC values calculated for trimethoprim, ciprofloxacin, moxifloxacin, and meropenem were seven-, four-, three-, and twofold higher than at neutral pH, respectively. Tobramycin was the only antibiotic with modestly improved activity at acidic pH. Our data suggest that the nutritional environment impacts *B. cenocepacia* susceptibility, and more specifically, that acidic pH decreases *B. cenocepacia* susceptibility to most of the antibiotics currently used to treat CF infections.

Since *B. cenocepacia* is intrinsically resistant to multiple antibiotics, CF patients are often treated with combinations of two or three antibiotics at the same time. El-Halfawy et al. (31) recently proposed multiple antibiotic combinations against *B. cenocepacia* (31). These antibiotic combinations were used against *B. cenocepacia* in Luria Broth (LB) medium and include antibiotics like ceftazidime and moxifloxacin, shown to have decreased activity in SCFM-FeZn at acidic pH in our susceptibility assays. We assessed the activity of some of these antibiotic combinations in the SCFM-FeZn at acidic and neutral pH using checkerboard assays (Fig. S2). We tested ceftazidime in combination with moxifloxacin and colistin methanesulfonate, as well as trimethoprim in combination with moxifloxacin. We calculated the fractional inhibitory concentration index for these antibiotic combinations based on the fluorescence of our reporter strain (Table S2). None of the antibiotic combinations reported were synergistic in the SCFM-FeZn at either a neutral or acidic pH.

### Screening the Sweet library against *B. cenocepacia* in the SCFM-FeZn at acidic and neutral pH

**FIG 3:**
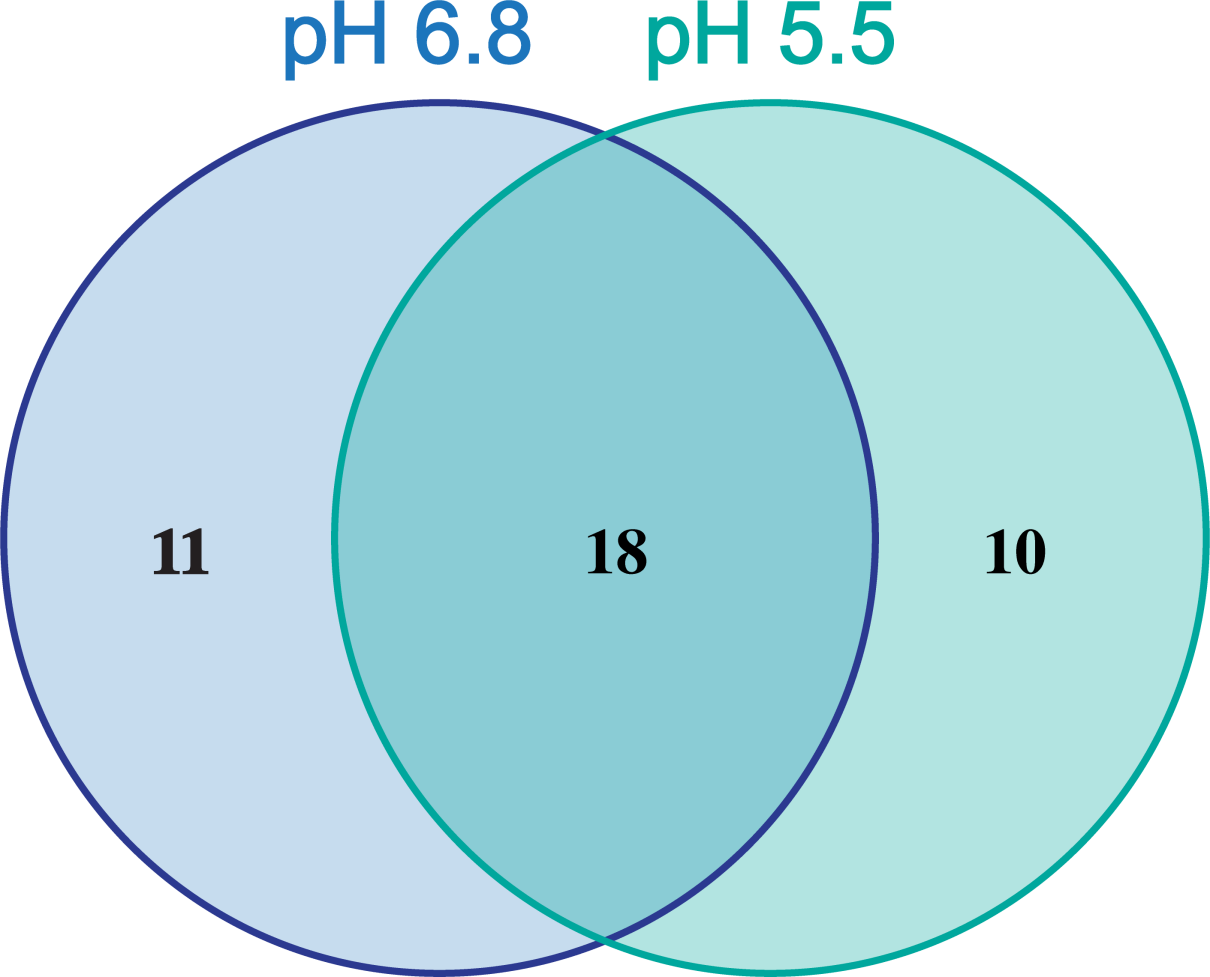
*B. cenocepacia* antimicrobial susceptibility to Sweet library compounds. SCFM-FeZn agar plates at pH 5.50 and 6.80 were inoculated with *B. cenocepacia* K56-2. Antibiotics were dispensed into the plates using a pinning robot. The plates were incubated overnight at 37°C, and inhibition zones were measured after 24 hours. Each compound was pinned in duplicate at each pH during two independent experiments, for a total of four replicates (*n* = 4).

Since pH impacted *B. cenocepacia* susceptibility to antimicrobials and antimicrobial combinations, we used a compound library to compare *B. cenocepacia* susceptibility to a large set of antibiotics at pH 5.5 and 6.8 in the SCFM-FeZn. The Sweet library is a custom compound library with 591 compounds, including most of the commonly used antibiotics to treat *B. cenocepacia* infections (28). We used a high-throughput screening robot to dispense the compounds into SCFM-FeZn agar plates previously inoculated with *B. cenocepacia* K56-2. Inhibition zones were measured after 24 hours of incubation. Out of 591 compounds present in the library, 39 were active against *B. cenocepacia* K56-2 (Fig. 3). Eighteen compounds were active at both pH values (Table S3). Eleven compounds were uniquely active at pH 6.8, and 10 were uniquely active at pH 5.5 (Table S4). Interestingly, none of the antibiotics most commonly used to treat CF infections were active against *B. cenocepacia* in the SCFM-FeZn agar plates. Out of the 18 compounds that were active at both pH values against *B. cenocepacia* in the SCFM-FeZn agar plates, nine were reproducibly active among all replicates. Novobiocin had the largest inhibition zone at acidic pH, and coumermycin A1 had the largest inhibition zone at neutral pH.

Four antibiotic compounds (coumermycin A1, novobiocin, mitomycin C, and streptonigrin) were selected based on reproducibility on agar plates and host-associated toxicity to measure MIC in liquid media. The MICs of these compounds (Table 1) were lower than those observed for antibiotics used most commonly to treat CF infections. When comparing MIC values at different pH values, novobiocin and streptonigrin had improved activity at acidic pH and coumermycin at neutral pH. There was no significant difference in the MIC of mitomycin C at neutral and acidic pH.

**TABLE 1:** *B. cenocepacia* susceptibility to selected compounds from the Sweet library in SCFM-FeZn at pH 5.50 and 6.80 (*n* = 4)^*a*^

Compound	MIC (μg/mL)		Significance
	pH 6.80	pH 5.50	
Novobiocin	1.29 ± 0.11	0.22 ± 0.04	***
Coumermycin A1	0.5 ± 0.11	6.21 ± 1.53	*
Mitomycin C	1.36 ± 0.25	1.98 ± 0.07	NS
Streptonigrin	16.94 ± 2.02	3.06 ± 1.14	*

^*a*^ *, *P* < 0.05; ***, *P* < 0.001; NS, *P* > 0.05.

## DISCUSSION

Here, we assessed the impact of acidic pH and increased iron and zinc concentrations on *B. cenocepacia* growth and susceptibility to antimicrobials in a medium that mimics the nutritional environment of the CF sputum (SCFM-FeZn). In addition, we compared growth and antimicrobial susceptibility to a rich medium like MH, often used for susceptibility testing in clinical settings. We first examined growth at an acidic pH in the CF nutritional context and found that *B. cenocepacia* grows faster in SCFM-FeZn at acidic pH than in MH and to higher cell density than in SCFM and SCFM-FeZn at neutral pH. Multiple studies showed that the pH values of CF airways are more acidic than those of healthy airways (10, 32), and in some cases, exhaled breath condensate acidification can be an indication of inflammation as well as bacterial exacerbations (33). Interestingly, *Burkholderia* species have increased abundance in acidic soils compared to more neutral or alkaline soils and are cultured at pH ranges between 4.0 and 6.0 in LB, Marine Broth (MB), or Agrobacterium (AB) minimal media (34 – 36). The ability of *B. cenocepacia* to thrive in acidic environments could be an advantage during infection in the CF context, and we hypothesize that acidity contributes to CF patients’ susceptibility to *Burkholderia* sp. infections.

In SCFM-FeZn, designed to mimic the CF environment, acidity reduces the efficacy of many antibiotics used to treat *B. cenocepacia* infection. In our experiments, aztreonam and ceftazidime fully lost their activity against *B. cenocepacia* at an acidic pH. Both antibiotics are beta-lactams known to inhibit peptidoglycan biosynthesis. Interestingly, previous studies reported that ceftazidime had activity at an acidic pH of 5.0 against uropathogenic strains of *Escherichia coli*, *Klebsiella pneumoniae*, *Proteus mirabilis*, *Enterococcus faecalis*, *Staphylococcus saprophyticus*, and *Staphylococcus epidermidis* (37). This suggests that the loss of activity of ceftazidime at an acidic pH is unique to *B. cenocepacia*. We also observed significantly decreased susceptibility of meropenem, moxifloxacin, ciprofloxacin, and trimethoprim at acidic pH. Similarly, decreased activity of moxifloxacin and ciprofloxacin was observed against *S. aureus* at an acidic pH (38, 39). Ciprofloxacin had decreased activity against Gram-negative bacteria such as *E. coli* and *P. aueruginosa* at an acidic pH (40). Also, the activity of trimethoprim against both Gram-negative and Gram-positive bacteria (*E. faecalis*, *Klebsiella oxytoca*, *P. mirabilis*, *S. aureus*, *and E. coli*) decreased at an acidic pH (41). In our assays, tobramycin was the only antibiotic with increased activity at acidic pH. The activity of tobramycin at multiple pH values has been assessed against *E. coli*, *K. pneumoniae*, *P. mirabilis*, *E. faecalis*, *S. saprophyticus*, and *S. epidermidis* and showed improved activity at neutral pH (7.0) compared to acidic pH (5.0–6.0) (37). This suggests that the increased activity of tobramycin at an acidic pH is unique to *B. cenocepacia* in the CF nutritional environment. Interestingly, most of the antibiotics currently used to treat *B. cenocepacia* infections in the CF context have decreased activity against Gram-positive and Gram-negative bacteria at an acidic pH.

We further assessed *B. cenocepacia* susceptibility to a library of 591 different compounds and found that novobiocin, coumermycin A1, mitomycin C, and streptonigrin are active against *B. cenocepacia* in the SCFM-FeZn at neutral and acidic pH. None of these antibiotics are currently used to treat *B. cenocepacia* infections. Novobiocin and coumermycin A1 act as antibiotics and anticancer agents that target DNA synthesis by inhibiting topoisomerase IV and the DNA gyrase (42, 43). In our assays, novobiocin had improved activity against *B. cenocepacia* at acidic pH. Loutet et al. (44) showed synergy of novobiocin in combination with polymixin B and colistin against *B. cenocepacia* (44). Low MIC values for novobiocin have been reported against *Burkholderia pseudomallei* and *Burkholderia mallei*. The MIC values for novobiocin against *B. pseudomallei* and *B. mallei* were 1.63 and 1.30 µg/mL in MH broth, respectively (45). In terms of its increased activity at acidic pH, Pomares et al. (46) showed that *Salmonella* Typhimurium susceptibility to novobiocin increases at acidic pH and suggested that acidic pH modifies the outer membrane permeability to novobiocin (46). This antibiotic was commercialized and approved for use in humans in the 1950s, mostly to treat Gram-positive infections. Novobiocin was delisted after the development of resistance and reports that some patients treated with novobiocin developed a severe rash (47, 48).

On the other hand, coumermycin A1 was discovered after novobiocin and demonstrated greater antibacterial potency but had low oral bioavailability and aqueous solubility as well as high irritability (48). Coumermycin was also shown to be effective against *Burkholderia* species and to have superior properties than novobiocin. Willcocks et al. (49) found that coumermycin has increased activity against *B. pseudomallei* in *G. mellonella* larvae compared to novobiocin and protected against acute melioidosis in a murine infection model (49). They attributed increased protection to a longer half-life in plasma compared to novobiocin.

Mitomycin C was another compound active against *B. cenocepacia*, and its MIC values were not significantly different between pH values in SCFM-FeZn. Mitomycin C is an antitumor and antibacterial agent that crosslinks DNA strands (50). In 2020, the US Food and Drug Administration approved mitomycin C for treating patients with urothelial cancer. There are reports of mitomycin C being effective against persister cells of *Acinetobacter baumannii*, *E. coli*, *S. aureus*, and *P. aeruginosa* (51, 52). No reports of antibiotic activity at different pH values were found; however, studies reported that mitomycin C was more cytotoxic to cancer cells at an acidic pH (53). In addition, we found that streptonigrin was active against *B. cenocepacia* at neutral and acidic pH, with enhanced activity at acidic pH. Streptonigrin is a metal-binding antibiotic from *Streptomyces flocculus* (54). Streptonigrin has both antibiotic and antitumor activities by releasing reactive oxygen radicals and inhibiting RNA and DNA syntheses (55). Interestingly, the activity of streptonigrin is linked to the presence of metal ions such as zinc, copper, iron, manganese, cadmium, and gold (55). White and Yeowell (56) showed that the bactericidal effect of streptonigrin against *E. coli* is enhanced by the presence of iron (56). Cohen et al. (57) also showed iron-dependent killing of *Neisseria gonorrhoeae* by streptonigrin (57). Perhaps the increased activity of this compound at an acidic pH in the SCFM-FeZn is linked to the increased solubility of iron in the media.

All in all, we have shown that acidic pH increases *B. cenocepacia* growth in the CF nutritional environment. *B. cenocepacia* susceptibility in MH to some antibiotics was significantly different compared to SCFM-FeZn at neutral pH. However, acidic pH significantly decreased *B. cenocepacia* susceptibility to most of the commonly used antibiotics tested. Our findings give a possible explanation for the poor relationship between *in vitro* susceptibility and clinical outcome and support the use of media that better mimic the environment at the site of infection for susceptibility testing. We also found four active compounds at both pH values in the CF nutritional environment. Future research could be directed toward improving these antibiotics’ formulation and administration against *B. cenocepacia* infections in CF.

## SUPPLEMENTAL MATERIAL

Supplemental material spectrum.02731-23-s0001.docx Fig. S1 and S2 and Tables S1 to S4.

ASM does not own the copyrights to Supplemental Material that may be linked to, or accessed through, an article. The authors have granted ASM a non-exclusive, world-wide license to publish the Supplemental Material files. Please contact the corresponding author directly for reuse.
