# Supplementary material for: Acidic pH modulates Burkholderia cenocepacia antimicrobial susceptibility in the cystic fibrosis nutritional environment
Source: Microbiol Spectr. 2023 Nov 15;11(6):e02731-23. doi: 10.1128/spectrum.02731-23 (PMC10714822; doi:10.1128/spectrum.02731-23)
Supplement: Supplemental material — Fig. S1 and S2 and Tables S1 to S4. [file spectrum.02731-23-s0001.docx]

**Supplemental material**

**Acidic pH modulates *Bukholderia cenocepacia* antimicrobial susceptibility in the cystic fibrosis nutritional environment**

L. Daniela Morales^1^, Yossef Av-Gay^1,2^, Michael E. P. Murphy^1^

^1^Department of Microbiology and Immunology, Life Sciences Institute, The University of British Columbia, Vancouver, British Columbia, Canada.

^2^Department of Medicine, Division of Infectious Diseases, The University of British Columbia, Vancouver, British Columbia, Canada.


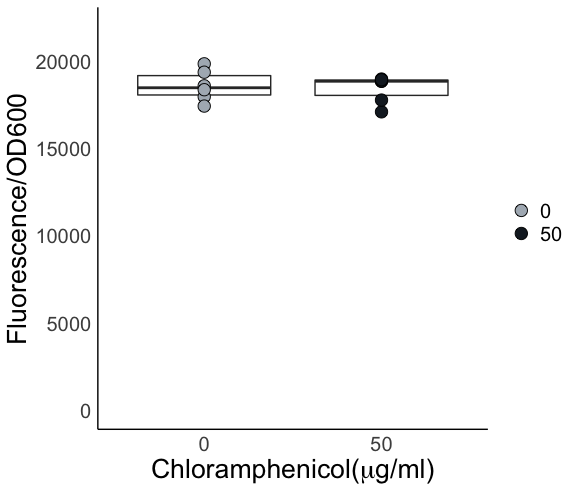


**Fig S1*. B. cenocepacia* K56-2 reporter strain fluorescence stability.** *B. cenocepacia* K56-2 reporter strain was grown on 100 μl of Mueller Hinton Broth supplemented with or without 50 µg/ml of chloramphenicol (Cm50) for antibiotic selection. Fluorescence (λ_Ex_: 470 nm, λ_Em_: 510 nm) and OD_600_ were measured after 16 hours. A paired T-test was performed fluorescence values (*p*-value =0.72). Data reported as mean from two independent experiments with three biological replicates each (n = 6).


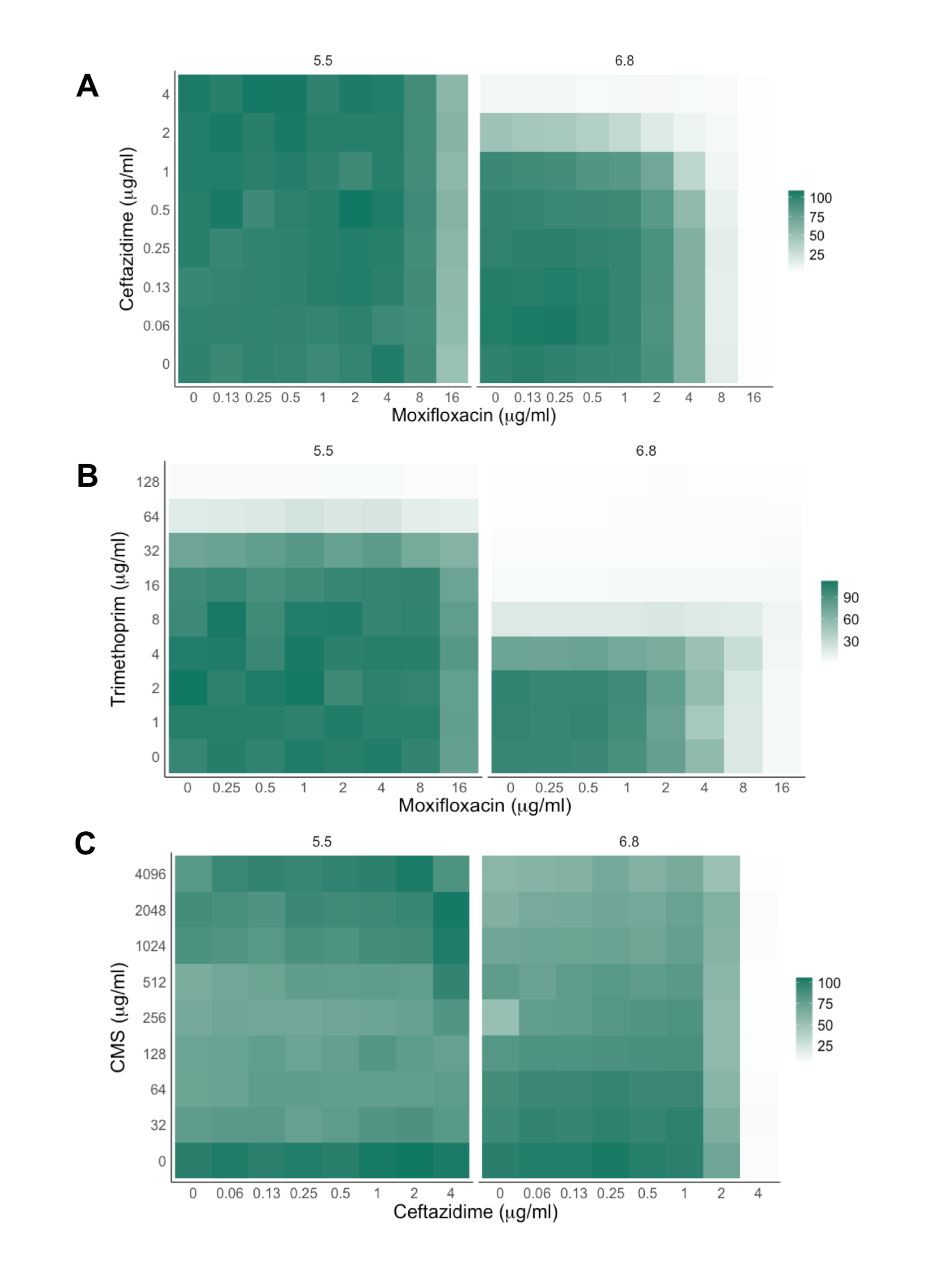


**Fig. S2. *B. cenocepacia* susceptibility to antibiotic combinations.** *B. cenocepacia* reporter strain was used for checkerboard assays in 96 well plates: (**A**) ceftazidime and moxifloxacin, (**B**) trimethoprim and moxifloxacin, and (**C**) colistin methanesulfonate (CMS) and ceftazidime. Cells were incubated for 16 hours at 37 ºC. Growth percentage was calculated by interpreting the fluorescence in the absence of antibiotics as 100% growth. Values shown represent the average growth of four biological replicates (n = 2 - 4).

**Table S1.  *B. cenocepacia* growth rate in SCFM-FeZn at pH 5.50 and 6.80 compared to MH and SCFM (n=6).**

| **Media** | **Maximum growth rate**  **(Fluorescence(AU)/h)** |
| --- | --- |
| MH | 622 ± 46 |
| SCFM | 750 ± 30 |
| SCFM-FeZn pH 5.50 | 1014 ± 18 |
| SCFM-FeZn pH 6.80 | 687 ± 14 |

**Table S2. Fractional inhibitory concentration indices (FICI) for antibiotic combinations against *B. cenocepacia* K56-2 in SCFM-FeZn.**

|  | **Average FICI** | |
| --- | --- | --- |
| **Antibiotic combinations** | **pH 6.8** | **pH 5.5** |
| Ceftazidime + moxifloxacin | 1.69 ± 0.97 | ND |
| Trimethoprim + moxifloxacin | 1.45 ± 0.05 | ND |
| CMS + ceftazidime | ND | ND |

(ND) Not determined. (CMS) colistin methanesulfonate.

Table S3. Inhibition zones of sweet library compounds active against *B. cenocepacia* at acidic and neutral pH in the SCFM-FeZn (n=4).

|  | **pH 5.5** | | **pH 6.8** | |
| --- | --- | --- | --- | --- |
| **Compound^1^** | **Inhibition zone (mm)** | **SD** | **Inhibition zone (mm)** | **SD** |
| Alexidine⁺ | 1.0 | 0.0 | 1.0 | 0.0 |
| BAY 11-7082 | 0.25 | 0.50 | 1.50 | 1.73 |
| Bismuth(III) | 1.75 | 2.36 | 1.50 | 1.73 |
| Bithionol | 0.25 | 0.50 | 0.25 | 0.50 |
| CCCP⁺ | 1.25 | 0.50 | 0.50 | 0.58 |
| Chlortetracycline | 0.50 | 0.58 | 0.50 | 0.58 |
| Coumermycin A-1⁺ | 1.25 | 0.50 | 13.00 | 0.00 |
| Ebselen | 0.25 | 0.50 | 0.25 | 0.50 |
| Echinomycin | 0.75 | 0.50 | 0.50 | 0.58 |
| Mitomycin C⁺ | 1.00 | 1.41 | 4.50 | 4.04 |
| Nadifloxacin | 0.50 | 0.58 | 0.50 | 0.58 |
| Novobiocin⁺ | 11.50 | 1.73 | 2.00 | 2.31 |
| Oxolinic acid⁺ | 3.50 | 0.58 | 2.50 | 1.73 |
| Patulin | 0.75 | 0.96 | 0.50 | 0.58 |
| Phenylmercuric acetate⁺ | 2.50 | 0.58 | 2.25 | 0.96 |
| Streptonigrin⁺ | 2.00 | 0.82 | 1.50 | 1.00 |
| Thimerosal⁺ | 6.00 | 2.94 | 3.50 | 0.58 |
| Tyrothricin⁺ | 1.00 | 0.00 | 1.00 | 0.00 |

^1^Compounds in blue had larger average inhibition zones at neutral pH and compounds in green had larger average inhibition zones at acidic pH.

SD = Standard deviation

⁺Compounds that were active in all replicates.

Table S4 Inhibition zones of sweet library compounds active against *B. cenocepacia* at either pH in the SCFM-FeZn (n=4)

| **pH 5.5** | | |
| --- | --- | --- |
| **Compound** | **Inhibition zone (mm)** | **SD** |
| Actinomycin D | 0.25 | 0.50 |
| Bithionol sulfoxide⁺ | 1.00 | 0.00 |
| Chloromercuribenzoic acid⁺ | 1.00 | 0.00 |
| Flurbiprofen | 2.00 | 2.45 |
| Fusidic acid | 0.25 | 0.50 |
| Gallium(III)ProtoporphyrinIX | 0.25 | 0.50 |
| Minocycline | 2.00 | 4.00 |
| Nigericin⁺ | 1.50 | 0.58 |
| Stigmatellin | 0.25 | 0.50 |
| Tetracycline | 2.00 | 2.45 |
| **pH 6.8** | | |
| **Compound** | **Inhibition zone (mm)** | **SD** |
| 1,4-Naphthoquinone | 0.50 | 0.58 |
| 2-Mercaptopyridine N-oxide | 0.50 | 0.58 |
| Alamethicin | 0.25 | 0.50 |
| BAY 11-7085 | 0.25 | 0.50 |
| Broxyquinoline | 0.25 | 0.50 |
| Deferoxamine | 0.50 | 0.58 |
| Dequalinium | 0.50 | 0.58 |
| Dibromothymoquinone | 0.25 | 0.50 |
| Hydroxypyridine-2-thione zinc salt | 2.00 | 2.31 |
| Meclocycline | 0.50 | 0.58 |
| Piericidin A | 0.50 | 0.58 |

(SD) Standard deviation

(⁺) Compounds that were active in all replicates.
